# The influence of racism on cigarette smoking: Longitudinal study of young people in a British multiethnic cohort

**DOI:** 10.1371/journal.pone.0190496

**Published:** 2018-01-24

**Authors:** Ursula M. Read, Alexis Karamanos, Maria João Silva, Oarabile R. Molaodi, Zinat E. Enayat, Aidan Cassidy, J. Kennedy Cruickshank, Seeromanie Harding

**Affiliations:** 1 School of Population Health Sciences & School of Life Course Sciences, Kings College London, London, United Kingdom; 2 ESRC International Centre for Lifecourse Studies in Society and Health, Department of Epidemiology and Health, University College London, London, United Kingdom; 3 MRC/CSO Social and Public Health Sciences Unit, Institute of Health and Wellbeing, University of Glasgow, Glasgow, Scotland; 4 University College London Hospitals, National Hospital for Neurology and Neurosurgery, Queen Square London, United Kingdom; Queensland University of Technology, AUSTRALIA

## Abstract

**Introduction:**

Studies, predominantly from the US, suggest that positive parenting, social support, academic achievement, and ethnic identity may buffer the impact of racism on health behaviours, including smoking, but little is known about how such effects might operate for ethnically diverse young people in the United Kingdom. We use the Determinants of young Adult Social well-being and Health (DASH), the largest UK longitudinal study of ethnically diverse young people, to address the following questions: a) Is racism associated with smoking? b) Does the relationship between racism and smoking vary by gender and by ethnicity? (c) Do religious involvement, parenting style and relationship with parents modify any observed relationship? and d) What are the qualitative experiences of racism and how might family or religion buffer the impact?

**Methods:**

The cohort was recruited from 51 London schools. 6643 were seen at 11-13y and 4785 seen again at 14-16y. 665 participated in pilot follow-up at 21-23y, 42 in qualitative interviews. Self-report questionnaires included lifestyles, socio-economic and psychosocial factors. Mixed-effect models examined the associations between racism and smoking.

**Results:**

Smoking prevalence increased from adolescence to age 21-23y, although ethnic minorities remained less likely to smoke. Racism was an independent longitudinal correlate of ever smoking throughout adolescence (odds ratio 1.77, 95% Confidence Interval 1.45–2.17) and from early adolescence to early 20s (1.90, 95% CI 1.25–2.90). Smoking initiation in late adolescence was associated with cumulative exposure to racism (1.77, 95% CI 1.23–2.54). Parent-child relationships and place of worship attendance were independent longitudinal correlates that were protective of smoking. Qualitative narratives explored how parenting, religion and cultural identity buffered the adverse impact of racism.

**Conclusions:**

Racism was associated with smoking behaviour from early adolescence to early adulthood, regardless of gender, ethnicity or socio-economic circumstances adding to evidence of the need to consider racism as an important social determinant of health across the life course.

## Introduction

The United Kingdom (UK), like many high-income countries, has seen significant decreases in tobacco smoking among adolescents [1]. However inequalities have increased with smoking initiation highest among the economically disadvantaged [1–3]. Adolescence is a vulnerable period for the initiation of health risk behaviours which can have enduring adverse effects in adulthood. In Britain, 58% of heavy smokers started smoking regularly before age 16 [2]. Early uptake of smoking is associated with heavier tobacco use in adulthood, higher dependency, and greater risk of morbidity and mortality [4]. Factors influencing smoking initiation among young people operate at individual, social and structural levels and include parental/sibling smoking [5], peer influence [1], mental health [6], media exposure [7] and tobacco marketing [7]. In the UK girls are more likely to smoke than boys [1]. It is difficult to make robust inferences about age trends in smoking among UK ethnic minorities but the few existing studies show significant differences by gender. At ages 16-64y, smoking prevalence appears to be higher among Black Caribbean and Bangladeshi men than men from other ethnic groups and lower among South Asian and Black African women than among White British and Black Caribbean women [8]. Although deprivation accounts for ethnic differences in smoking for men, this does not operate in the same way for women [8].

These findings point to possible socio-cultural influences on smoking behaviour. However most studies on ethnicity and smoking in the UK lack the power to discriminate between groups such as Black Caribbeans and Black Africans who have differing cultural norms and migration histories. Whilst cultural or religious norms may proscribe smoking for some groups, particularly for girls, aspects of ‘acculturation’ and peer pressure may erode these influences [9]. On the other hand cultural integration and aspects of what has been called ‘enculturation’ or ‘ethnic socialisation’ may have benefits for mental health, adaptation, and health behaviours [10–13].

Adverse childhood experiences and stressful life events, such as experiences of racism and discrimination, have been associated with increased smoking among adolescents [14, 15]. Research suggests that racism-related stress may trigger physiological, psychological and behavioural responses including the use of alcohol, tobacco and drugs [16–19], but is relatively unexplored in the UK and in young people. We use racism as a social construct which refers to various manifestations of oppression and discrimination based on perceptions of difference related to concepts of nationality, ethnicity, culture and religion [20]. Racism has been characterised as interpersonal (direct experiences such as insults or physical assault), internalised (incorporation of ideologies about the inferiority of one's own ethnic group), and institutional or systemic (embedded in social structures such as policies and social norms) [20, 21]. Within an ecological model racism and discrimination therefore act as determinants of health inequalities through multiple intersecting pathways [22–24]. Longitudinal studies conducted predominantly with adult samples, including a recently published study from the UK [25], provide evidence that whilst there may be a role for stress in appraisal of events as racist or discriminatory, experiences of racism precede stress. Consistent with the ‘weathering hypothesis’, these studies argue that culmulative exposure to racism over time, and in different contexts, has deleterious effects on health over the life-course [26–28].

The rise in attacks against ethnic and religious groups in the UK [29] in the context of inequality, austerity, terrorism, increasing migration and ‘Brexit’ make addressing the consequences for health an important public health concern. Anti-immigrant rhetoric as well as the exclusionary effect of policy changes may produce additional stress for ethnic minority groups, with accompanying impacts on health [30]. Studies suggest that racism is associated with health inequalities, including poor mental health, across all ethnic groups [16]. However, several studies have shown that ethnic minority adolescents in the UK report better mental health compared to White British [31, 32], despite more reported racism and deprivation. These findings, as well as lower rates of smoking for some ethnic groups, raise questions as to what might protect young people, particularly those from disadvantaged backgrounds, from engaging in health risk behaviours.

Research on protective factors for young people has largely drawn on theories of resilience [33], and social capital [34]. Socio-ecological models propose that resilience, defined as ‘positive adaptation in the face of adversity’ [33], is developed through the interaction between psychological, social, cultural and environmental factors such as identity, family life, religion and neighbourhoods [35]. Importantly, resilience requires not only the availability of resources, but opportunities to access them [36]. Social capital, i.e. access to social networks and resources, has been theorised to operate through 'bonding capital' within groups or ‘bridging capital’ across groups [34]. A recent review found that positive parent-child relations and engagement in family activities were protective against tobacco use in young people, and some evidence of a protective effect from religious attendance [37]. Studies suggest that positive parenting, social support, academic achievement, religion and ethnic identity may buffer the impact of discrimination on adolescent mental health and health behaviours, though evidence is mixed and precisely how such factors may buffer the effects of racism on health is unexplored [11, 38–42]. In line with theories of resilience and bonding social capital, experiences of discrimination may strengthen group solidarity and attachment to positive group values which work to counter the negative effects. Parents and religious groups, for example, may provide positive messages about ethnic identity which counter stigmatising attitudes and promote adhesion to cultural or religious ideals [39]. At the same time, mixing with other ethnic groups (cultural integration) may enhance bridging social capital and open opportunities, such as for educational achievement, which may in turn buffer the effects of racism and promote healthy behaviours [43].

Much of the research on racism and health risk behaviours has been conducted with African Americans and US Hispanics and there are few longitudinal studies [16]. Little is known about how these effects might operate in the UK with different ethnic groups, migration histories and social and political context. There are also limitations in the extent to which quantitative studies can investigate how such buffering effects may operate in particular contexts. Quantitative surveys may under-estimate experiences of racism or discrimination [44] and may not capture all the salient aspects of individual experience, particularly for disadvantaged or minority groups [45, 46]. Qualitative inquiry can provide deeper and more nuanced exploration of how racism or discrimination is perceived and interpreted, the particularities of context, and the diverse ways in which family and social environments may enhance coping and resilience [44, 45, 47, 48]. Qualitative studies suggest that aspects of tradition, culture, religion and family life play an important role in cultivating social norms and values which operate in different ways to heighten or mitigate health risk behaviours among young people [43] but there are very few published studies from the UK.

In this paper we use findings from the Determinants of young Adult Social well-being and Health (DASH), the largest UK longitudinal study of ethnically diverse young people, to address the following questions: a) Is racism associated with smoking? b) Does the relationship between racism and smoking vary by gender and by ethnicity? (c) Do religious involvement, parenting style and relationship with parents modify any observed relationship? d) What are the qualitative experiences of racism and discrimination and how might family or religion buffer the impact in an ethnically diverse context?

## Methods

### Sample

The DASH sample was recruited between 2002 and 2003 from 51 schools in 10 London boroughs. Details of the study are described elsewhere [49]. A total of 6643 students, aged 11-13y, took part in the baseline survey. We take the position that ethnic identity is dynamic and multidimensional, reflecting historic social and cultural traditions and current context [50]. Ethnicity in DASH was measured by self-report utilising over 25 ethnic categories derived from the British Census, including options for ‘mixed’ and ‘other’. Separate questions asked about country of birth of self, parents and grandparents. Self-ascribed ethnicity was compared with these responses to check for inconsistencies. There was also a separate question on religious affiliation. Eighty per cent were from ethnic minorities including Indian, Pakistani, Bangladeshi, Black African, Black Caribbean and mixed ethnicity. For analysis, Bangladeshis and Pakistanis were combined because of relatively small sample sizes. Both groups were distinct from Indians in that they were more economically disadvantaged and predominantly Muslim. In 2005–06 4782 (88% of children in 49 schools, 72% of the cohort), aged 14-16y, took part in the first follow-up. In 2012–14 a 10% subsample (N = 665, ~100/major ethnic group representative by gender and SEC), aged 21-23y, took part in a pilot follow-up study, 42 of whom took part in qualitative interviews.

### Quantitative measures

All measures used here were captured by self-complete questionnaires. Two binary response variables were created for smoking. Ever-smoked was based on a binary response of ‘no’ or ‘yes’ to “Have you ever smoked a cigarette?” Initiation of smoking after 11-13y was defined as never smoked at 11-13y and smoking regularly or occasionally at 14-16y (combined to binary response ‘no’ or ‘yes’). Reported racism/discrimination (‘no’ or ‘yes’) was assessed using the experiences of discrimination scale which includes questions on 'unfair treatment' on the grounds of race, skin colour, place of birth and religion in various locations e.g. school, work, on the street [51]. These are: ‘Has anyone made you feel bad or hassled you because of your race, skin colour, or where you were born?’ and ‘Has anyone made you feel bad or hassled you because of your religion?’

Generational status was defined as being born in the UK or not. Cultural integration was derived from questions regarding friendships—integrated (friendships with own and with other ethnic groups), traditional (friendships mainly with own ethnic group), assimilated (friendships mainly with other ethnic groups) and marginalised (friendships with neither own nor other ethnic groups) [10]. Questions on parenting included self-reported quality of the relationship with a key parent and perceived parenting style using the Parental Bonding Instrument (PBI) [52]. Two variables—'care' and 'control'—were derived in tertiles. Questions on religion included religious affiliation and frequency of attendance to a place of worship. The Strengths and Difficulties Questionnaire was used to measure psychological well-being [53]. A Total Difficulties Score was derived with higher scores indicating greater difficulties. A cut-off point of >17 was used to identify potentially clinically-significant cases. The General Health Questionnaire (GHQ-12) [54] was used at 21-23y with a score of ≥4 indicating psychological distress. Measures of individual SEC in adolescence included the Family Affluence Scale (FAS) [55], based on number of cars, computers and holidays, family type, and parental employment. At 21-23y, SEC was measured using own employment.

### Qualitative interviews

Qualitative interviews were informed by grounded theory [56], with the aim to deepen conceptual and contextual analysis of multidimensional measures used in the questionnaires, including measures of discrimination.

Sampling: Stratified purposeful sampling was used to achieve broad representation across socio-demographic criteria (gender, ethnicity, religion, family type, SEC). Interviews were conducted by UR, a White British female with a background in in UK NHS mental health services in London and a PhD in anthropology. She has clinical and research experience in West Africa and with ethnic minorities in the UK. Contact was established by telephone, SMS or face-to-face when participants attended for physical measures. Reasons for conducting the interview were explained verbally and in an information sheet given to participants prior to the interview.

Data collection: Interviews were conducted in participants’ homes or at Kings College London. Interviews were conducted by UR alone with the participant. Forty-two participants took part, broadly distributed across ethnic groups and gender except for female Indians (see Table 1). There were few refusals. Reasons for refusal, including from female Indians, were mainly because potential participants reported they were too busy with study/work. Interviews were semi-structured using a topic guide developed by UR and SH (see S1 Appendix). Topics covered present circumstances and recall of experiences in adolescence including family/peer relationships, ethnicity, religion and identity, and stressful life events and responses, including experiences of racism or discrimination. Interviews were digitally recorded and lasted 45 minutes-2 hours. Notes were taken immediately following the interview to record relevant observations e.g. participants’ demeanour, appearance and communication style. If the interview was conducted at the participant’s home written observations were also recorded on aspects of the neighbourhood and home environment.

**Table 1:** Qualitative interview participants.

Ethnicity	Male	Female
White British	3	3
Black Caribbean	4	4
Black African	3	9
Indian	3	0
Bangladeshi	1	3
Pakistani	3	2
Other	2	2
Total	19	23

### Analysis

Three-level logistic mixed models with measurements nested within pupils and schools were used the full cohort follow-up at 14-16y. Two-level logistic mixed models with measurements nested within pupils were used for those followed up at 21-23y. Age (yearly) was linearly associated with ever smoked and smoking initiation. The models described below used variables that were statistically related to smoking in univariate analyses. All variables were considered as time-dependent, except generational status, gender and ethnicity. Model 0 examined the unadjusted association between self-reported racism and ever smoked or smoking initiation in adolescence, while Model 1 examined the association between self-reported racism and ever smoked or smoking initiation, after taking into account gender, age, ethnicity, and generational status. Model 2 controlled additionally for religious affiliation, religious attendance, cultural integration, psychological distress, parental smoking, relationship with key parent, parental control and parental care. Model 3 controlled additionally for SEC. Model 4 (final model) included variables that were significant in Model 3, after applying Wald tests. Interaction terms explored whether racism effects varied by age or ethnicity. The same modelling approach examined the influence of racism on smoking from 11-13y to 21-23y. Multiple imputation by chained equations [57] was used to handle missing data on covariates. All analyses were conducted using STATA 13 and statistical significance was considered as -*p*<0.05.

Qualitative interviews were transcribed verbatim by a professional transcription company and checked against the audio recordings by UR. Interviews were analysed iteratively alongside quantitative findings to provide context for questionnaire responses e.g. narrative accounts of experiences of racism, meanings/behaviours attributed to religious affiliation, and to inform interpretation of how quantitative variables might modify the racism-smoking relationship. Transcripts were coded by UR using NVivo 10. Broad first level codes were developed by UR and SH from *a priori* themes derived from the questionnaire. Drawing on grounded theory, a process of constant comparison [56] was employed to identify emerging inductive themes and develop sub-codes which were refined as coding progressed. A selection of transcripts was independently coded by two authors (UR/SH). Divergences were discussed to reach consensus and coding refined.

### Ethics

Approval for the study was obtained from the South East Scotland Research Ethics Committee and from Local Education Authorities when participants were at school. Parents were provided with information packs prior to the start of the study, via head teachers, and a parental opt-out consent procedure was followed. Written informed consent was obtained from all participants.

## Results

Table 2 shows the profile of the sample by ethnicity at ages 11–13 years. There was a pattern of lower proportions of those who had ever smoked among ethnic minority groups than their White British peers, but this was significant only for Indian males and females and Black African females. All ethnic minority groups reported higher proportions of racism, significant only for Pakistani/Bangladeshi males, compared with their White British peers.

**Table 2:** Sample profile at 11-13y by gender and ethnicity. The Determinants of Adolescent (now Adult) Social well-being and Health study.

	White UK (N = 873)		Black Caribbean (N = 779)		Black African (N = 892)		Indian (N = 419)		Pakistani and Bangladeshi (N = 446)		Other (N = 1373)	
	Males (N = 492)	Females (N = 381)	Males (N = 390)	Females (N = 389)	Males (N = 417)	Females (N = 475)	Males (N = 237)	Females (N = 182)	Males (N = 306)	Females (N = 140)	Males (N = 772)	Females (N = 601)
Ever smoked	25.6 (21.7 to 30.0)	26.4 (22.1 to 31.2)	22.8 (18.2 to 28.2)	28.9 (24.3 to 34.1)	19.5 (15.4 to 24.4)	13.8 (10.7 to 17.8)*	9.6 (6.0 to 14.9)*	6.9 (3.8 to 12.5)*	20.2 (15.6 to 25.6)	12.2 (15.6 to 25.6)	22.7 (19.5 to 26.2)	24.6 (19.5 to 26.2)
Reported racism^a^	14.0 (11.2 to 17.4)	12.6 (9.6 to 16.3)	13.6 (10.5 to 17.4)	18.5 (14.9 to 22.7)	19.4 (15.9 to 23.5)	17.7 (14.5 to 21.4)	18.1 (13.7 to 23.6)	20.9 (15.6 to 27.4)	27.8 (23.0 to 33.1)*	25.7 (19.1 to 33.6)	21.6 (18.9 to 24.7)	17.5 (14.6 to 20.7)
**Psychosocial factors**												
*Parenting*												
Getting on very well with key parent	79.3 (75.4 to 82.6)	73.7 (69.1 to 77.9)	77.4 (72.7 to 81.6)	75.5 (70.8 to 79.6)	74.3 (69.9 to 78.3)	71.8 (67.6 to 75.7)	74.7 (68.7 to 79.8)	68.7 (61.5 to 75.0)	77.8 (72.6 to 82.1)	75.0 (67.1 to 81.5)	74.9 (71.7 to 77.8)	67.1 (63.2 to 70.7)
Getting on quite well with key parent	15.2 (12.3 to 18.7)	18.9 (15.3 to 23.2)	20.5 (16.6 to 25.2)	21.6 (17.7 to 26.0)	11.8 (9.0 to 15.2)	20.0 (16.6 to 23.8)	10.5 (7.2 to 15.2)	16.5 (11.7 to 22.6)	10.8 (7.8 to 14.8)	14.3 (9.4 to 21.2)	15.0 (12.7 to 17.7)	21.6 (18.5 to 25.1)
Getting on not so well with key parent	1.2 (0.5 to 2.7)	3.7 (2.2 to 6.1)	2.1 (1.0 to 4.2)	3.0 (1.6 to 5.3)	2.2 (1.1 to 4.1)	3.2 (1.9 to 5.2)	-	0.5 (0.1 to 3.8)	1.3 (0.5 to 3.4)	2.9 (1.1 to 7.4)	2.2 (1.4 to 3.5)	5.3 (3.8 to 7.4)
High parental care tertile^b^	37.6 (34.5 to 43.2)	45.4 (40.5 to 51.5)	36.4 (31.8 to 41.3)	38.0 (33.3 to 43.0)	35.5 (31.0 to 40.2)	35.2 (31.0 to 39.6)	39.7 (33.6 to 46.0)	37.9 (31.1 to 45.2)	38.9 (33.6 to 44.5)	40.0 (32.2 to 48.4)	39.5 (36.1 to 43.0)	33.6 (29.9 to 37.5)
Intermediate parental care tertile^b^	34.3(30.3 to 38.9)	30.4 (26.0 to 35.3)	20.5 (16.8 to 24.8)*	24.4 (20.4 to 29.0)	23.3 (19.4 to 27.6)*	26.9 (23.1 to 31.1)	24.0 (19.0 to 29.9)*	28.6 (22.4 to 35.6)	29.1 (24.2 to 34.4)	25.7 (19.1 to 33.6)	27.7 (24.7 to 31.0)	30.1 (26.6 to 33.9)
Low parental care tertile^b^	25.0 (21.4 to 29.0)	22.0 (18.2 to 26.5)	33.1(28.6 to 37.9)	29.3 (25.0 to 34.0)	29.7 (25.5 to 34.3)	30.9 (26.9 to 35.3)	30.8 (25.2 to 37.0)	30.2 (24.0 to 37.3)	27.8 (23.0 to 33.1)	30.0 (23.0 to 39.1)	26.0 (23.1 to 29.2)	28.9 (25.5 to 32.7)
Low parental control tertile^b^	38.8 (34.5 to 43.3)	46.5 (41.5 to 51.6)	33.5 (28.8 to 38.8)	26.3 (21.8 to 30.9)*	22.3 (18.3 to 26.8)*	21.7 (18.1 to 25.9)*	24.6 (19.3 to 30.6)*	22.7 (17.1 to 29.5)*	19.1 (15.0 to 24.0)*	22.2 (16.0 to 30.1)*	24.2 (21.2 to 27.5)*	21.8 (18.5 to 25.4)*
Intermediate parental control tertile	40.5 (36.2 to 45.0)	37.4 (32.6 to 42.4)	35.0 (30.2 to 40.2)	35.5 (30.8 to 40.7)	38.0 (33.2 to 43.1)	35.0 (30.7 to 39.6)	36.2 (30.1 to 42.7)	42.6 (35.5 to 50.1)	39.9 (34.4 to 45.7)	34.1 (26.5 to 42.5)	42.4 (38.9 to 46.1)	40.5 (36.5 to 44.6)
High tertile parental control tertile^b^	20.7 (17.3 to 24.6)	16.1 (12.7 to 20.2)	31.5 (26.7 to 36.4)*	38.2 (33.4 to 43.5)*	39.7 (34.8 to 44.8)*	43.3 (38.7 to 48.0)*	39.2 (33.1 to 45.9)*	34.7 (28.0 to 42.0)*	41.0 (35.4 to 46.7)*	43.7 (35.5 to 52.2)*	33.4 (20.0 to 36.9)	37.7 (33.8 to 41.9)*
Religion												
Non Catholic Christianity	28.3 (24.4 to 32.4)	29.9 (25.5 to 34.7)	56.1 (51.2 to 61.0)	61.2 (56.2 to 65.9)	40.5 (35.9 to 45.3)	41.9 (37.5 to 46.4)	3.4 (1.7 to 6.6)	7.1 (4.2 to 11.9)	0.6 (0.02 to 2.6)	-	22.5 (19.7 to 25.6)	28.3 (24.8 to 32.0)
Catholicism	11.4 (8.9 to 14.5)	16.0 (12.7 to 20.1)	10.8 (8.0 to 14.3)	19.5 (15.9 to 23.8)	15.3 (12.2 to 19.1)	27.4 (23.5 to 31.6)	2.1 (0.9 to 5.0)	5.5 (3.0 to 9.9)	0.3 (0.04 to 0.2)	-	15.2 (12.8 to 17.9)	21.5 (18.4 to 24.9)
Hinduism	-	-					19.4 (14.8 to 25.0)	16.5 (11.7 to 22.6)	86.3 (81.9 to 89.7)	88.6 (82.1 to 92.9)	24.5 (21.6 to 27.6)	16.0 (13.3 to 19.1)
Islam	0.8 (0.3 to 2.2)	0.3 (0.01 to 1.8)	-	-	23.5 (19.7 to 27.8)	14.9 (12.0 to 18.5)	45.1 (39.9 to 51.6)	48.9 (41.7 to 56.2)	0.3 (0.04 to 2.3)	-	1.3 (0.7 to 2.4)	1.0 (0.4 to 2.2)
Other	15.4 (12.5 to 18.9)	11.8 (8.9 to 15.5)	24.1 (20.1 to 28.6)	12.1 (9.2 to 15.7)	18.7 (15.2 to 22.7)	13.5 (10.7 to 16.9)	17.3 (13.0 to 22.7)	15.4 (10.8 to 21.4)	12.1 (8.9 to 16.3)	11.4 (7.1 to 17.9)	18.4 (15.8 to 21.3)	14.8 (12.2 to 17.9)
None	42.7 (38.4 to 47.1)	41.5 (36.6 to 46.5)	7.2 (5.0 to 10.2)	5.4 (3.5 to 8.1)	1.2 (0.5 to 2.9)	0.6 (0.2 to 1.9)	0.8 (0.2 to 3.3)	0.5 (0.1 to 3.8)	-	-	12.3 (10.2 to 14.8)	11.6 (9.3 to 14.5)
*Religious attendance*												
More than once a week	5.9 (4.1 to 8.5)	9.4 (6.8 to 12.9)	32.3 (27.8 to 37.1)	45.8 (40.9 to 50.7)	59.5 (54.7 to 64.1)	70.3 (66.0 to 74.3)	44.7 (38.5 to 51.1)	40.7 (33.7 to 38.0)	69.0 (63.5 to 73.9)	45.7 (37.6 to 54.1)	27.3 (24.3 to 30.6)	27.1 (23.7 to 30.8)
Less than once a week	27.6 (23.7 to 31.9)	35.1 (30.3 to 40.2)	36.9 (32.3 to 41.8)	34.4 (29.9 to 39.2)	18.9 (15.5 to 23.0)	15.4 (12.4 to 18.9)	34.2 (28.4 to 40.5)	38.5 (31.6 to 45.8)	16.3 (12.6 to 20.9)	19.3 (13.5 to 26.7)	30.2 (27.0 to 33.5)	34.6 (30.9 to 38.5)
Never	66.5 (62.0 to 70.7)	55.5 (50.3 to 60.6)	12.8 (9.8 to 16.5)*	13.6 (10.6 to 16.5)*	5.8 (3.9 to 8.5)*	5.1 (3.4 to 7.4)*	4.6 (2.6 to 8.2)*	7.1 (4.2 to 11.9)*	2.9 (1.5 to 5.6)*	22.9 (16.6 to 30.6)*	31.3 (28.2 to 34.7)*	29.8 (26.3 to 33.6)*
Generational status												
Born in the UK	97.7 (95.9 to 98.7)	97.6 (95.5 to 98.8)	74.4 (70.0 to 78.5)	73.5 (68.9 to 77.7)	51.3 (46.5 to 56.1)	63.6 (59.1 to 67.8)	81.7 (76.1 to 86.2)	78.6 (72.0 to 84.0)	78.1 (73.1 to 82.5)	86.1 (79.2 to 91.0)	64.5 (61.0 to 67.8)	69.3 (65.4 to 72.9)
Born abroad	2.3 (1.3 to 4.1)	2.4 (1.2 to 4.5)	23.1 (19.2 to 27.5)	22.9 (19.0 to 27.3)	44.6 (39.9 to 49.4)	33.9 (29.8 to 38.3)	18.3 (13.8 to 23.9)	21.4 (16.0 to 28.0)	21.9 (17.5 to 26.9)	13.9 (9.0 to 20.8)	35.5 (32.2 to 39.0)	30.7 (27.1 to 34.6)
*Cultural integration*^*c*^												
Integrated	28.5 (24.6 to 32.8)	29.4 (24.9 to 34.2)	27.4 (23.2 to 32.1)	29.3 (25.0 to 34.0)	29.5 (25.3 to 34.1)	27.6 (23.7 to 31.8)	34.4 (28.0 to 41.3)	24.7 (18.5 to 32.0)	36.0 (30.4 to 42.0)	22.7 (16.2 to 30.8)	26.0 (22.9 to 29.4)	23.4 (20.0 to 27.1)
Traditional	35.9 (31.6 to 40.3)	32.1 (27.5 to 37.1)	31.0 (26.6 to 35.8)	36.0 (31.3 to 40.9)	17.3 (13.9 to 21.2)*	25.1 (21.3 to 29.2)	26.2 (20.4 to 32.8)	24.7 (18.5 to 32.0)	32.6 (27.2 to 38.5)	25.0 (18.2 to 33.3)	18.2 (15.5 to 21.2)*	20.3 (17.1 to 23.8)*
Assimilated	17.9 (14.7 to 21.7)	21.4 (17.5 to 26.0)	11.8 (8.9 to 15.4)	11.8 (9.0 to 15.4)*	23.3 (19.4 to 27.6)	26.5 (22.7 to 30.7)	21.0 (15.8 to 27.4)	31.0 (24.3 to 38.7)	18.9 (14.6 to 24.1)	29.7 (22.4 to 28.2)	36.8 (33.2 to 40.5)*	37.2 (33.3 to 41.4)*
Marginalised	17.7 (14.5 to 21.5)	17.0 (13.5 to 21.3)	12.8 (9.8 to 16.5)	15.9 (12.6 to 19.9)	12.7 (8.8 to 16.3)	13.3 (10.5 to 16.6)	18.5 (13.6 to 24.6)	19.6 (14.1 to 26.6)	12.5 (9.0 to 17.0)	22.7 (16.2 to 30.8)	19.0 (16.3 to 22.2)	19.2 (16.1 to 22.7)
*Psychological distress*^*d*^												
Total Difficulties Score^c^	15.9 (12.9 to 19.4)	15.5 (12.2 to 19.5)	15.6 (12.3 to 19.6)	17.5 (14.0 to 21.6)	14.1 (11.1 to 17.8)	15.6 (12.6 to 19.1)	16.5 (12.2 to 21.8)	10.4 (6.7 to 15.8)	11.8 (8.6 to 15.9)	19.3 (13.5 to 26.7)	13.5 (11.3 to 16.1)	15.8 (13.1 to 19.0)
**Socio economic circumstances**												
*Family structure and parental employment*												
2 parent family, both parents employed	67.5 (63.2 to 71.5)	64.8 (59.9 to 69.5)	47.2 (42.2 to 52.2)*	38.0 (33.3 to 43.0)*	48.2 (43.4 to 53.0)*	54.7 (50.2 to 59.2)	67.1 (60.8 to 72.8)	74.7 (67.8 to 80.5)	59.2 (53.5 to 64.5)	57.1 (48.8 to 65.1)	51.4 (47.8 to 54.9)*	47.0 (43.0 to 51.0)*
1 parent family, 1 parent employed	13.8 (11.0 to 17.2)	13.6 (10.5 to 17.5)	24.9 (20.8 to 29.4)*	37.0 (32.03 to 41.9)	15.6 (12.4 to 19.4)	17.3 (14.1 to 10.9)	2.5 (1.1 to 5.5)*	3.8 (1.8 to 7.8)*	2.0 (0.9 to 4.3)*	0.7 (0.01 to 4.9)*	12.6 (10.4 to 15.2)	16.5 (13.7 to 19.7)
2 parent family, both parents unemployed	4.7 (3.1 to 6.9)	4.7 (3.0 to 7.4)	1.6 (0.7 to 3.4)	2.1 (1.0 to 4.1)	5.3 (3.5 to 7.9)	6.7 (4.8 to 9.4)	13.1 (9.3 to 18.0)*	6.0 (3.4 to 10.6)	18.0 (14.0 to 22.7)*	25.7 (19.1 to 33.6)*	12.4 (10.2 to 14.9)*	13.7 (11.1 to 16.7)*
1 parent family, parent unemployed	7.3 (5.3 to 10.0)	10.8 (8.0 to 14.3)	12.3 (9.4 to 16.0)	14.4 (11.2 to 18.3)	13.2 (10.3 to 16.8)*	12.6 (9.9 to 15.9)	2.1 (0.9 to 5.0)	1.6 (0.5 to 5.0)	8.5 (5.8 to 12.2)	7.9 (4.4 to 13.7)	13.5 (11.3 to 16.1)*	14.8 (12.2 to 17.9)*
Other family type	1.2 (0.5 to 2.7)	1.6 (0.7 to 3.5)	2.8 (1.6 to 5.2)	4.6 (2.9 to 7.2)	3.6 (2.2 to 5.9)	3.2 (1.9 to 5.2)	0.4 (0.1 to 3.0)	0.5 (0.1 to 3.8)	0.1 (0.03 to 3.0)	-	2.3 (1.5 to 3.7)	2.3 (1.4 to 3.9)
*Family Affluence Scale*^*e*^												
High family affluence	68.1 (63.6 to 72.2)	71.9 (66.9 to 76.3)	48.2 (43.3 to 53.2)*	43.3 (38.6 to 48.4)*	46.5 (41.8 to 51.3)*	50.5 (46.0 to 55.0)*	67.4 (60.3 to 73.7)	62.4 (54.3 to 69.9)	66.3 (60.3 to 71.9)	45.3 (36.5 to 54.4)*	62.0 (58.2 to 65.6)	55.0 (50.7 to 59.3)*
Intermediate family affluence	26.7 (22.8 to 31.1)	24.7 (20.5 to 29.5)	26.4 (22.3 to 31.0)	33.9 (29.5 to 38.8)*	27.1 (23.0 to 31.6)	30.9 (26.9 to 35.5)	30.5 (24.4 to 37.5)	34.2 (27.0 to 42.3)	30.3 (25.0 to 36.1)	50.4 (41.4 to 59.4)*	32.1 (28.6 to 35.7)	39.1 (35.0 to 43.4)*
Low family affluence	5.2 (3.5 to 7.7)	3.4 (1.9 to 5.9)	4.4 (2.7 to 6.0)	5.9 (4.0 to 8.7)	2.9 (1.6 to 5.0)	2.7 (1.6 to 4.7)	2.1 (0.8 to 5.5)	3.4 (1.4 to 7.9)	3.4 (1.8 to 6.5)	4.3 (1.8 to 9.9)	6.0 (4.4 to 8.1)	5.8 (4.1 to 8.2)
*Parental smoking*												
Maternal smoking	35.9 (31.5 to 40.5)	37.0 (32.1 to 42.2)	15.1 (11.9 to 19.0)*	23.1 (19.2 to 27.6)*	2.4 (1.43 to 4.4)*	1.9 (1.0 to 3.6)*	1.0 (0.3 to 4.0)*	1.9 (0.6 to 5.6)*	2.3 (1.0 to 5.1)*	1.7 (0.4 to 6.5)*	27.5 (24.0 to 31.1)*	26.8 (23.1 to 31.0)*
Paternal smoking	32.8 (28.4 to 37.6)	32.4 (27.5 to 37.7)	32.0 (26.5 to 38.0)	31.4 (26.2 to 37.1)	10.1 (7.2 to 14.1)*	9.9 (7.3 to 13.4)*	17.2 (12.5 to 23.1)*	18.9 (13.5 to 25.8)*	31.6 (26.2 to 37.6)	31.1 (23.4 to 40.0)	35.2 (31.4 to 39.2)	31.9 (27.7 to 36.3)

Note: Not all percentages add up to 100% due to missing values

*p<0.05 indicates different compared with White British boys/girls

^a^ Experiences of discrimination scale which includes questions on 'unfair treatment' on the grounds of race, skin colour, place of birth and religion in various locations e.g. school, work, on the street [51]

^b^ Perceived parental care and control measured using the Parental Bonding Instrument [52]

^c^ Responses to questions about friendships with peers of the respondent’s own or other ethnic group were used to measure cultural integration. Based on their responses participants were classified as integrated (friendships with own and with other ethnic groups), traditional (friendships only with own ethnic group), assimilated (friendships only with other ethnic groups) and marginalized (friendships with neither own nor the dominant other ethnic group) [10]

^d^ Total Difficulties Score derived from the Strengths and Difficulties Questionnaire [53]. Score of >17 indicates psychological distress/behavioural difficulties

^e^ Family Affluence Scale derived from number of cars or vans, computers, and holidays [55]

Table 3 shows the profile of the sample by ethnicity at 14–16 years. The proportions that ever smoked increased across all ethnic groups, but generally remained lower among ethnic minority groups than White British. Smoking initiation between 11–13 years and 14–16 years was also generally lower among ethnic minority groups, significant for Black Caribbean, Black African and Indian males and among all ethnic minority groups for females. Smoking initiation was lower among Black Africans than Black Caribbeans. At age 14–16 years, all ethnic minority males, except Pakistani and Bangladeshi males, and all ethnic minority females were significantly more likely to report racism compared with their White British peers, with significant increases between 11–13 years and 14–16 years for some groups, notably Black Africans.

**Table 3:** Sample profile at 14-16y by gender and ethnicity. The Determinants of Adolescent (now Adult) Social well-being and Health study.

	White UK (N = 873)		Black Caribbean (N = 779)		Black African (N = 892)		Indian (N = 419)		Pakistani and Bangladeshi (N = 446)		Other (N = 1373)	
	Males (N = 492)	Females (N = 381)	Males (N = 390)	Females (N = 389)	Males (N = 417)	Females (N = 475)	Males (N = 237)	Females (N = 182)	Males (N = 306)	Females (N = 140)	Males (N = 772)	Females (N = 601)
Ever smoked	51.1 (46.7 to 55.6)**	65.3 (60.4 to 70.0)**	36.5 (31.8 to 41.5)*/**	55.3 (50.3 to 60.2)*/**	27.1 (23.0 to 31.6)*	31.9 (27.8 to 36.3)*/**	24.9 (19.7 to 30.9)*/**	27.5 (21.4 to 34.4)*/**	39.0 (33.6 to 44.6)8/**	33.8 (26.4 to 42.1)*/**	43.3 (39.8 to 46.9)	51.1 (47.1 to 55.1)*/**
Initiated smoking	15.3 (12.1 to 19.1)	28.5 (24.0 to 33.5)	6.7 (4.3 to 10.5)*	13.1 (9.8 to 17.3)*	2.8 (1.4 to 5.5)*	5.2 (3.3 to 8.0)*	6.9 (3.9 to 11.8)*	4.2 (1.9 to 9.0)*	11.8 (8.3 to 16.5)	1.7 (0.4 to 6.7)*	10.3 (8.1 to 13.1)	15.3 (12.4 to 18.8)*
Reported racism^a^	20.3 (17.0 to 24.0)	16.3 (12.9 to 20.3)	28.8 (24.5 to 33.6)*/**	28.3 (24.0 to 33.0)*/**	33.1 (28.7 to 37.8)*/**	33.3 (29.2 to 37.6)*/**	32.1 (26.4 to 38.3)*/**	29.7 (23.5 to 36.7)*/**	26.1 (21.5 to 31.4)	31.4 (24.2 to 39.6)*	27.6 (24.5 to 30.9)*	32.3 (28.7 to 36.1)*/**
**Psychosocial factors**												
*Parenting*												
Getting on very well with key parent	68.3 (64.0 to 72.2)**	62.2 (57.2 to 66.9)**	28.3 (23.9 to 33.0)*/**	38.3 (33.5 to 43.3)*/**	70.3 (65.7 to 74.5)	53.5 (49.0 to 57.9)*/**	76.4 (70.5 to 81.4)	70.3 (63.3 to 76.5)	76.1 (71.0 to 80.6)	65.7 (57.4 to 73.1)	69.6 (66.2 to 72.7)	55.4 (51.4 to 59.3)**
Getting on quite well with key parent	28.9 (25.0 to 33.0)	28.6 (24.3 to 33.4)	48.5 (43.5 to 53.6)*	46.3 (41.4 to 51.4)*	24.5 (20.6 to 28.8)	33.3 (29.2 to 37.6)	21.9 (17.1 27.7)	24.7 (19.0 to 31.5)	20.3 (16.1 to 25.2)	29.3 (22.3 to 37.4)	24.3 (21.4 to 27.5)	33.9 (30.3 to 37.8)
Getting on not so well with key parent	2.6 (1.5 to 4.5)	8.1 (5.8 to 11.3)	5.0 (3.2 to 7.7)	11.5 (8.7 to 15.1)*	3.8 (29.2 to 37.6)	10.9 (8.4 to 14.1)*/**	1.3 (0.4 to 3.9)	4.9 (2.6 to 9.3)	1.6 (0.7 to 3.9)	5.0 (2.4 to 10.2)	5.6 (4.1 to 7.4)**	9.5 (7.4 to 12.1)**
High parental care tertile^b^	26.2 (22.5 to 30.3)	23.4 (19.4 to 28.0)	23.5 (19.5 to 28.1)	15.3 (12.0 to 19.3)	22.1 (18.3 to 26.3)	17.3 (14.1 to 20.9)	31.2 (25.6 to 37.4)	25.8 (20.0 to 32.7)	29.1 (24.2 to 34.4)	26.4 (19.7 to 34.4)	25.8 (22.8 to 29.0)	21.3 (18.2 to 24.8)
Intermediate parental care tertile^b^	29.9 (26.0 to 34.1)	31.9 (27.4 to 36.8)	27.0 (22.7 to 31.7)	26.5 (22.3 to 31.1)	27.1 (23.0 to 31.6)	24.0 (20.4 to 28.1)	30.4 (24.8 to 36.6)	26.9 (21.0 to 33.9)	29.4 (24.6 to 24.8)	18.6 (12.9 to 26.0)	29.5 (26.4 to 32.9)	23.3 (20.1 to 26.8)
Low parental care tertile^b^	44.0 (39.6 to 48.4)**	44.7 (39.7 to 49.8)**	49.5 (44.3 to 54.5)**	58.2 (53.2 to 63.0)*/**	47.5 (42.7 to 52.3)**	56.8 (52.3 to 61.2)*/**	38.0 (32.0 to 44.3)	46.7 (39.5 to 54.0)**	39.9 (34.5 to 45.5)**	54.3 (45.9 to 62.4)**	43.9 (40.4 to 47.4)**	54.2 (50.2 to 58.2)**
Low parental control tertile^b^	47.4 (43.0 to 51.9)	44.0 (39.0 to 49.1)	33.9 (29.2 to 38.9)*	26.2 (22.1 to 30.9)*	26.0 (21.9 t 30.5)*	23.2 (19.6 to 27.3)*	27.2 (21.0 to 33.3)*	23.6 (18.0 to 30.4)*	23.6 (19.1 to 28.7)*	20.7 (14.8 to 28.3)*	30.5 (27.3 to 33.9)*	25.6 (22.2 to 29.3)*
Intermediate parental control tertile	34.3 (30.3 to 38.7)	33.1 (28.5 to 38.0)	37.6 (32.8 to 42.6)	31.9 (27.5 to 36.8)	37.6 (33.0 to 42.5)	28.6 (24.7 to 32.9)	36.6 (30.6 to 43.0)	31.9 (25.5 to 39.0)	39.2 (33.8 to 44.9)	27.9 (21.0 to 35.9)	35.3 (32.0 to 38.8)	31.3 (27.7 to 35.2)**
High tertile parental control tertile^b^	18.2 (15.0 to 21.9)	22.9 (18.9 to 27.5)	28.6 (24.2 to 33.3)*	41.8 (37.0 to 46.8)*	36.4 (31.8 to 41.2)*	36.4 (31.8 to 41.2)*	36.2 (30.2 to 42.5)*	44.5 (37.4 to 51.8)*	37.2 (31.9 to 42.8)*	51.4 (43.1 to 59.6)*	34.2 (30.9 to 37.6)*	43.1 (39.2 to 47.1)*
Religion												
Non Catholic Christianity	25.6 (21.9 to 29.7)	26.5 (22.3 to 31.2)	60.3 (55.3 to 65.0)	63.5 (58.6 to 68.1)	46.8 (42.0 to 51.6)	50.7 (46.2 to 55.2)	4.2 (2.3 to 7.7)	6.0 (3.4 to 10.6)	0.7 (0.2 to 2.6)	-	20.3 (17.6 to 23.3)	26.0 (22.6 to 29.6)
Catholicism	10.8 (8.3 to 13.8)	16.8 (13.4 to 20.9)	13.6 (10.5 to 17.4)	19.8 (16.1 to 24.1)	20.1 (16.6 to 24.3)	27.6 (23.7 to 31.8)	3.0 (1.4 to 6.1)	5.0 (2.6 to 9.3)	-	-	21.2 (18.5 to 24.3)	23.1 (19.9 to 26.7)
Hinduism	0.02 (0.002 to 1.4)	-	-	-	-	-	22.4 (17.5 to 28.1)	18.1 (13.2 to 24.4)	95.8 (92.8 to 97.5)	97.9 (93.5 to 99.3)	25.1 (22.2 to 28.3)	15.8 (13.1 to 19.0)
Islam	0.8 (0.3 to 2.2)		1.3 (0.05 to 3.0)	0.8 (0.2 to 2.4)	26.1 (22.2 to 30.6)	16.2 (13.2 to 19.8)	55.3 (48.9 to 61.5)	63.2 (55.9 to 69.9)	0.3 (0.04 to 2.3)	-	1.2 (0.6 to 2.2)	0.8 (0.3 to2.0)
Other	6.3 (4.5 to 8.8)	6.8 (4.7 to 9.8)	7.4 (5.2 to 10.5)	5.7 (3.7 to 8.4)	3.1 (1.8 to 5.3)	2.7 (1.6 to 4.7)	11.4 (7.9 to 16.1)	7.1 (4.2 to 11.9)	-	-	13.2 (11.0 to 15.8)	14.6 (12.0 to 17.7)
None	53.2 (48.8 to 57.6)	46.2 (41.2 to 51.2)	13.8 (10.8 to 17.7)*	8.2 (5.9 to 11.4)*	0.7 (0.2 to 2.2)*	0.4 (0.1 to 1.7)*	0.8 (0.2 to 3.3)*	0.5 (0.1 to 3.8)*	0.7 (0.2 to 2.6)*	0.7 (0.1 to 4.9)*	16.2 (13.7 to 19.0)*	16.6 (13.9 to 19.8)*
*Religious attendance*												
More than once a week	4.8 (3.2 to 7.1)	7.4 (5.2 to 10.6)	27.2 (23.0 to 31.8)*	38.0 (33.3 to 43.0)*	68.1 (63.5 to 72.4)*	67.8 (63.4 to 71.8)*/**	42.6 (36.4 to 49.0)*	30.8 (24.5 to 37.9)*/**	73.5 (68.3 to 78.2)*	22.1 (16.0 to 29.8)*/**	26.0 (23.1 to 29.3)*	24.1 (20.9 to 27.7)**
Less than once a week	23.1 (19.5 to 27.1)	27.9 (23.6 to 32.7)	46.7 (41.8 to 51.6)**	45.8 (40.8 to 50.8)**	22.3 (18.6 to 26.6)	25.7 (21.9 to 29.8)**	48.5 (42.2 to 54.9)**	58.2 (50.9 to 65.2)**	20.9 (16.7 to 25.9)	42.1 (34.2 to 50.5)**	34.8 (31.6 to 38.3)	35.3 (31.5 to 39.2)
Never	72.1 (68.0 to 76.0)	64.6 (59.6 to 69.3)	22.3 (18.4 to 26.7)	15.2 (11.9 to 19.1)	6.9 (4.9 to 9.8)	4.4 (2.9 to 6.7)	7.1 (4.5 to 11.3)	11.0 (7.2 to 16.5)	3.3 (1.8 to 6.0)	34.3 (26.9 to 42.6)	36.8 (33.4 to 40.3)	38.4 (34.6 to 42.4)**
*Cultural integration*^*c*^												
Integrated	34.8 (30.7 to 39.1)	31.5 (27.0 to 36.3)	34.1 (29.6 to 39.0)	27.8 (23.5 to 32.4)	41.2 (36.6 to 46.0)**	30.5 (26.5 to 34.8)	44.1 (37.8 to 50.5)	35.7 (29.1 to 43.0)	47.8 (42.2 to 53.5)**	34.5 (27.1 to 42.9)	33.0 (29.7 to 36.4)	27.5 (24.0 to 31.3)
Traditional	32.9 (28.9 to 37.2)	33.1 (28.5 to 38.0)	35.9 (31.3 to 40.8)	43.4 (38.6 to 48.4)	18.9 (15.5 to 23.0)*	32.0 (27.9 to 36.3)	23.3 (18.3 to 29.2)	26.4 (20.5 to 33.3)	29.8 (24.8 to 35.2)	28.8 (21.8 to 36.9)	17.4 (14.9 to 20.3)*	18.3 (15.4 to 21.7)
Assimilated	19.1 (15.9 to 22.8)	22.6 (18.6 to 27.0)	11.3 (8.5 to 14.8)*	14.9 (11.7 to 18.8)	24.9 (21.0 to 29.3)	26.5 (22.7 to 30.7)	24.6 (19.5 to 30.5)	33.5 (27.0 to 40.7)*	15.4 (11.7 to 20.0)	26.6 (19.9 to 34.6)	37.3 (34.0 to 40.8)*	43.6 (39.7 to 47.7)*
Marginalised	12.6 (9.9 to 15.8)	11.5 (8.7 to 15.2)	15.4 (12.1 to 19.3)	12.3 (9.4 to 16.0)	12.2 (9.4 to 15.7)	9.5 (7.1 to 12.5)	8.1 (5.2 to 12.3)**	4.4 (2.2 to 8.6)*/**	7.0 (4.6 to 10.5)	10.1 (6.0 to 16.3)	12.3 (10.1 to 14.8)**	10.5 (8.3 to 13.3)**
*Psychological distress*^*d*^												
Total Difficulties Score^c^	10.0 (7.6 to 12.9)**	14.4 (11.2 to 18.3)	8.7 (6.3 to 12.0)**	13.1 (10.1 to 16.9)	7.7 (5.5 to 10.7)**	13.5 (10.7 to 16.7)	11.4 (7.9 to 16.1)	7.1 (4.2 to 11.9)	6.2 (4.0 to 9.5)	15.7 (10.5 to 22.8)	8.6 (6.8 to 10.8)**	16.7 (13.9 to 19.9)
**Socio economic circumstances**												
*Family structure and parental employment*												
2 parent family, both parents employed	59.3 (54.9 to 63.6)	58.5 (53.5 to 63.4)	46.2 (41.1 to 51.1)*	35.5 (30.9 to 40.4)	47.7 (42.9 to 52.5)*	50.3 (45.8 to 54.8)	77.6 (71.9 to 82.5)*	76.9 (70.2 to 82.5)	64.7 (59.2 to 70.0)	49.3 (41.0 to 57.6)	13.7 (11.4 to 16.3)	12.8 (10.4 to 15.8)
1 parent family, 1 parent employed	13.2 (10.5 to 16.5)	14.2 (11.0 to 18.1)	29.0 (24.7 to 33.7)	37.3 (32.6 to 42.2)	17.5 (14.1 to 21.5)	20.2 (16.8 to 24.1)	4.6 (2.6 to 8.2)	6.0 (3.4 to 10.6)	5.0 (3.0 to 8.0)	7.9 (4.4 to 13.7)	5.2 (3.8 to 7.0)	7.1 (5.4 to 9.5)
2 parent family, both parents unemployed	3.9 (2.5 to 6.0)	4.5 (2.8 to 7.1)	3.1 (1.8 to 5.3)	1.5 (0.7 to 3.4)	6.0 (4.1 to 8.7)	5.1 (3.4 to 7.4)	8.0 (5.2 to 12.2)	6.6 (3.8 to 11.3)	16.3 (12.6 to 20.9)*	22.9 (16.6 to 30.6)*	1.6 (0.9 to 2.7)	2.8 (1.8 to 4.5)
1 parent family, parent unemployed	2.8 (1.7 to 4.8)**	2.4 (1.2 to 4.4)**	5.6 (3.7 to 8.4)**	5.9 (4.0 to 8.7)**	10.8 (8.1 to 14.2)*	9.9 (7.5 to 12.9)*	3.0 (1.4 to to 6.1)	1.1 (0.03 to 11.3)	5.5 (3.5 to 8.8)	8.6 (4.9 to 14.5)	1.6 (0.9 to 2.7)	2.0 (1.1 to 3.5)
Other family type	3.3 (2.0 to 5.2)	3.9 (2.4 to 6.4)	-	-	-	0.2 (0.02 to 1.5)	-	-	8.5 (5.8 to 12.2)*/**	11.4 (7.1 to 17.9)*/**	-	-
*Family Affluence Scale*^*e*^												
High family affluence	69.6 (65.3 to 73.5)	75.5 (70.8 to 79.6)	61.8 (56.9 to 66.5)**	56.0 (51.0 to 60.9)**	64.5 (59.8 to 69.0)**	62.5 (58.1 to 66.8)**	74.6 (68.5 to 79.8)	74.0 (67.0 to 80.0)	75.5 (70.3 to 80.1)	58.1 (49.6 to 66.1)	68.9 (65.5 to 72.1)	62.6 (58.6 to 66.4)
Intermediate family affluence	27.8 (23.9 to 31.9)	22.9 (18.9 to 27.5)	29.5 (25.2 to 34.2)	38.6 (33.8 to 43.5)*	28.8 (24.6 to 33.3)	33.5 (29.4 to 37.9)*	24.6 (19.4 to 30.6)	26.0 (20.0 to 33.0)	23.8 (19.3 to 29.0)	41.2 (33.2 to 49.7)*	29.6 (26.5 to 33.0)	35.0 (31.2 to 39.0)*
Low family affluence	2.7 (1.6 to 4.6)	1.6 (0.7 to 3.6)	3.3 (1.9 to 5.7)	3.3 (1.9 to 5.7)	1.9 (1.0 to 3.8)	0.6 (0.2 to 1.9)	0.1 (0.02 to 3.5)	-	0.7 (0.1 to 2.7)	0.7 (0.1 to 5.0)	1.5 (0.8 to 2.6)**	2.4 (1.4 to 4.0)
*Parental smoking*												
Maternal smoking	33.5 (29.4 to 37.8)	39.2 (34.3 to 44.3)	19.5 (15.8 to 23.8)	26.2 (22.0 to 30.9)	2.2 (1.1 to 4.2)	2.8 (1.6 to 4.8)	0.4 (0.1 to 3.1)	1.1 (0.3 to 4.4)	3.0 (1.6 to 5.8)	2.3 (0.7 to 7.0)	26.5 (23.5 to 29.8)	23.0 (19.7 to 26.6)
Paternal smoking	32.0 (28.0 to 36.2)	30.5 (26.0 to 35.3)	25.5 (21.3 to 30.1)	25.6 (21.4 to 20.2)	8.4 (6.1 to 11.6)	9.3 (6.9 to 12.3)	16.4 (12.1 to 21.7)	19.4 (14.3 to 25.9)	27.3 (22.6 to 32.7)	29.0 (22.0 to 37.1)	32.7 (29.4 to 36.1)	26.4 (23.0 to 30.1)

Note: Not all percentages add up to 100% due to missing values

*p<0.05 indicates different compared with White British boys/girls

** indicate differences compared with 11-13y within the same gender and ethnic group

^a^ Experiences of discrimination scale which includes questions on 'unfair treatment' on the grounds of race, skin colour, place of birth and religion in various locations e.g. school, work, on the street [51]

^b^ Perceived parental care and control measured using the Parental Bonding Instrument [52]

^c^ Responses to questions about friendships with peers of the respondent’s own or other ethnic group were used to measure cultural integration. Based on their responses participants were classified as integrated (friendships with own and with other ethnic groups), traditional (friendships only with own ethnic group), assimilated (friendships only with other ethnic groups) and marginalized (friendships with neither own nor the dominant other ethnic group) [10]

^d^ Total Difficulties Score derived from the Strengths and Difficulties Questionnaire [53]. Score of >17 indicates psychological distress/behavioural difficulties

^e^ Family Affluence Scale derived from number of cars or vans, computers, and holidays [55]

Other key features compared with White British peers include the ethnic patterning of parental styles, with greater proportions of ethnic minorities in the high control and in the low parental care tertiles; 40% of White British reporting no religion compared with <20% of ethnic minority groups, with high attendance to a place of worship at least once per week, highest among Black Africans and Pakistani/Bangladeshi females; and greater socio-economic disadvantage in ethnic minority groups with the exception of Indians.

### Racism and smoking in adolescence

Table 4 shows that racism was an independent longitudinal correlate of *ever smoking* in adolescence, before adjusting for confounders (Model 0:Odds Ratio 1.85, 95% confidence interval 1.58 to 2.17), and after adjusting for socio-demographic (Model 1: 2.05, 1.67 to 2.51), psychosocial factors (Model 2: 1.82, 1.46 to 2.25), and for selected variables that remained statistically significant (Model 4: 1.77, 1.45 to 2.17), namely age, ethnicity, relationship with key parent, parenting, cultural integration, psychological distress, maternal smoking, and SEC. Religion and attendance to a place of worship were not included in Model 4 as they were not significant on adjustment for SEC (Model 3). There was no significant interaction between racism and ethnicity (0.98, 0.95–1.01, p = 0.37) and gender (1.11, 0.82–1.52, p = 0.50), suggesting that the racism effect did not vary by these variables. There was, however, a significant interaction between racism and age (0.85, 0.76–0.96, p = 0.011) in the final model, which suggested that the effect of racism on smoking was less strong as adolescents became older.

**Table 4:** Ever smoking at 11-16y: The influence of racism, ethnicity, parenting and religious involvement, socio-economic circumstances: Odds ratio (95% confidence interval). The Determinants of Adolescent (now Adult) Social well-being and Health study.

	Model 0		Model 1		Model 2		Model 3		Model 4	
		p-value	Model o+ demographics	p-value	Model 1+ parenting, religious involvement and other psycho-social factors	p-value	Model 2+Socio-economic circumstances	p-value	Final model	p-value
**Racism (vs. no racism)**^**a**^	1.85 (1.58 to 2.17)	<0.001	2.05 (1.67 to 2.51)	<0.001	1.82 (1.46 to 2.25)	<0.001	1.82 (1.47 to2.25)	<0.001	1.77 (1.45 to 2.17)	<0.001
**Relationship with key parent (vs. getting on very well)**										
Getting on quite well					1.48 (1.17 to 1.85)	0.001	1.48 (1.17 to 1.86)	0.001	1.55 (1.24 to 1.95)	<0.001
Getting on not so well					2.25 (1.44 to 3.53)	<0.001	2.21 (1.41 to 3.46)	0.001	2.31 (1.51 to 3.54)	<0.001
**Parental care (vs. high parental care)**^**b**^										
Intermediate parental care tertile					1.55 (1.22 to 1.97)	0.001	1.54 (1.21 to 1.96)	<0.001	1.55 (1.24 to 1.95)	<0.001
Low parental care tertile					2.00 (1.54 to 2.60)	<0.001	2.02 (1.55 to 2.63)	<0.001	2.11 (1.65 to 2.69)	<0.001
**Parental control (vs. low tertile parental control)**^**b**^										
Intermediate parental care tertile					0.90 (0.72 to 1.13)	0.379	0.90 (0.72 to 1.13)	0.381		
High parental control tertile					1.17 (0.91 to 1.50)	0.228	1.17 (0.91 to 1.51)	0.212		
**Religion (vs. Non Catholic Christianity)**										
Catholicism					1.03 (0.73 to 1.44)	0.879	1.03 (0.73 to 1.44)	0.871		
Hinduism					0.93 (0.63 to 1.38)	0.722	0.93 (0.63 to 1.38)	0.660		
Islam					0.56 (0.26 to 1.20)	0.137	0.53 (0.25 to 1.15)	0.110		
Other					0.88 (0.61 to 1.28)	0.513	0.88 (0.61 to 1.27)	0.508		
None					1.22 (0.86 to 1.72)	0.262	1.21 (0.86 to 1.71)	0.274		
**Religious attendance (vs. < 1/week)**										
Once/week					1.19 (0.93 to 1.53)	0.159	1.20 (0.94 to 1.54)	0.144		
Never					0.98 (0.72 to 1.35)	0.913	0.99 (0.72 to 1.36)	0.951		
**Cultural integration (vs. Integrated)**^**c**^										
Traditional					1.05 (0.83 to 1.33)	0.67	1.05 (0.83 to 1.33)	0.69	1.09 (0.87 to 1.37)	0.433
Assimilated					0.75 (0.59 to 0.96)	0.023	0.76 (0.59 to 0.97)	0.027	0.79 (0.62 to 0.99)	0.042
Marginalised					0.74 (0.54 to 1.00)	0.049	0.75 (0.55 to 1.02)	0.067	0.75 (0.56 to 1.01)	0.055
**Psychological distress (TDS ≥17 vs < 17)**^**d**^					2.75 (2.06 to 3.66)	<0.001	2.75 (2.06 to 3.66)	<0.001	2.83 (2.17 to 3.70)	<0.001
**Ethnicity (vs. White UK)**										
Black Caribbean			0.70 (0.49–1.00)	0.047	0.71 (0.47 to 1.05)	0.084	0.70 (0.47 to 1.04)	0.078	0.71 (0.49 to 1.02)	0.066
Black African			0.21 (0.14–0.31)	<0.001	0.28 (0.18 to 0.44)	<0.001	0.28 (0.18 to 0.43)	<0.001	0.27 (0.18 to 0.41)	<0.001
Indian			0.11 (0.06–0.17)	<0.001	0.21 (0.11 to 0.42)	<0.001	0.22 (0.11 to 0.44)	<0.001	0.15 (0.09 to 0.25)	<0.001
Pakistani/Bangladeshi			0.32 (0.21–0.50)	<0.001	0.50 (0.28 to 0.90)	0.021	0.51 (0.29 to 0.92)	0.026	0.44 (0.27 to 0.70)	0.001
Other			0.67 (0.49–0.92)	0.015	0.75 (0.53 to 1.06)	0.103	0.76 (0.54 to 1.09)	0.135	0.73 (0.53 to 1.00)	0.05
**Born abroad (vs. born UK)**			0.70 (0.53–0.92)	0.001	0.74 (0.55 to 0.99)	0.003	0.76 (0.57 to 1.02)	0.072		
**Family structure and parental employment (vs. 2 parent family, both parents employed)**										
1 parent family, 1 parent employed							1.16 (0.86 to 1.57)	0.339		
2 parent family, both parents unemployed							1.12 (0.75 to 1.67)	0.594		
1 parent family, parent unemployed							1.10 (0.73 to 1.64)	0.646		
Other family type							1.94 (0.67 to 5.56)	0.219		
**Family Affluence Scale**^**e**^ **(vs.High family affluence)**										
Intermediate family affluence							0.74 (0.60 to 0.93)	0.009	0.79 (0.65 to 0.97)	0.028
Low family affluence							0.75 (0.41 to 1.38)	0.355	0.79 (0.46 to 1.35)	0.386
**Maternal smoking (vs. no maternal smoking)**					2.35 (1.79–3.10)	<0.001	2.39 (1.81 to 3.15)	<0.001	2.39 (1.85 to 3.10)	<0.001
**Females (vs. males)**			1.81 (1.42–2.31)	<0.001	1.54 (1.20–1.98)	0.001	1.54 (1.20 to 1.98)	0.001	1.57 (1.23 to 1.99)	<0.001
**Age**			2.18 (2.04–2.33)	<0.001	2.10 (1.94–2.27)	<0.001	2.10 (1.94 to 2.29)	<0.001	2.03 (1.89 to 2.17)	<0.001

Model 0: No adjustment for covariates. Model 1: Gender + age + ethnicity+ generational status. Model 2: Model 1 + religious affiliation+ religious attendance +cultural integration + psychological distress+ relationship with key parent +parental control+ parental care+maternal smoking+paternal smoking. Model 3: Models 1 and 2 + family affluence + family structure and parental employment status. Model 4 (based on Wald tests for variables in model 3): age, ethnicity, gender, cultural integration, maternal smoking, psychological distress, relationship with the key parent, parental care and family assets.

^a^ Experiences of discrimination scale which includes questions on 'unfair treatment' on the grounds of race, skin colour, place of birth and religion in various locations e.g. school, work, on the street [51]

^b^ Perceived parental care and control measured using the Parental Bonding Instrument [52]

^c^ Responses to questions about friendships with peers of the respondent’s own or other ethnic group were used to measure cultural integration. Based on their responses participants were classified as integrated (friendships with own and with other ethnic groups), traditional (friendships only with own ethnic group), assimilated (friendships only with other ethnic groups) and marginalized (friendships with neither own nor the dominant other ethnic group) [10]

^d^ Total Difficulties Score derived from the Strengths and Difficulties Questionnaire [53]. Score of >17 indicates psychological distress/behavioural difficulties

^e^ Family Affluence Scale derived from number of cars or vans, computers, and holidays [55]

Table 5 shows the effect of racism, derived to show exposure at either ages (baseline survey at 11-13y or follow-up survey at 14-16y) or cumulatively at both ages, on *smoking initiation* between 11-13y and 14-16y. Reported racism at both ages was consistently associated with smoking initiation, unadjusted for confounders (Model 0: odds ratio 1.59, 95% confidence interval 1.17 to 2.17), and after adjustment for demographic (Model 1: 2.09, 1.52–2.88) and psychosocial (Model 2: 1.68, 1.14–2.46) factors, and for variables that remained statistically significant (Model 4: 1.77, 1.23–2.54). There was also a suggestion of a (non-significant) gradient in each model, with odds ratios lowest for racism reported at 11-13y, highest for racism reported at both waves, and intermediate for reported racism at 14-16y. Religion was not included in model 4 as its effect was removed on adjustment for attendance to a place of worship (Model 2). For example, the lower likelihood of smoking initiation associated with being Muslim (0.86, 0.33 to2.24), and higher likelihood associated with not having a religion (1.22, 0.82 to 1.83) compared with non-Catholic Christians in the univariate analyses was no longer evident. As with ever smoking, interactions between racism and ethnicity (1.06, 0.98–1.14, p = 0.173) or gender (0.94, 0.61–1.44, p = 0.763) were not significant, and there was a significant interaction between reported racism and place of worship attendance on smoking initiation (2.25, 1.21 to 4.19, p = 0.011) in the final model. Adolescents who reported racism and never attending a place of worship were more likely to start smoking compared to those who reported racism and attending a place of worship more than once a week.

**Table 5:** Smoking initiation at 14-16y: The influence of racism, ethnicity, parenting and religious involvement, socio-economic circumstances: Odds ratio (95% confidence interval). The Determinants of Adolescent (now adult) Social well-being and Health study.

	Model 0		Model 1		Model 2		Model 3		Model 4	
		p-value	Model 0+ demographic characteristics	p-value	Model 1+ parenting, religious involvement and other psychosocial factors	*p-value*	Model 2+ Socio-economic circumstances	*p-value*	Final model	p-value
**Racism (vs. no racism)**^**a**^										
Racism at wave 1 only (11-13y)	1.26 (0.90 to 1.75)	0.172	1.45 (1.03 to 2.04)	0.032	1.28 (0.86 to 1.90)	0.225	1.35 (0.90 to 2.00)	0.143	1.40 (0.97 to 2.02)	0.07
Racism at wave2 only (14-16y)	1.41 (1.06 to 1.86)	0.018	1.63 (1.21 to 2.18)	0.001	1.37 (0.98 to 1.92)	0.067	1.38 (0.98 to 1.93)	0.066	1.50 (1.09 to 2.06)	0.013
Racism at both waves (11-13y and 14-16y)	1.59 (1.17 to 2.17)	0.003	2.09 (1.52 to 2.88)	<0.001	1.68 (1.14 to 2.46)	0.008	1.73 (1.18 to 2.55)	0.005	1.77 (1.23 to 2.54)	0.002
**Relationship with key parent at 14-16y (vs. getting on very well)**										
Getting on quite well					1.22 (0.91 to 1.63)	0.176	1.18 (0.88 to 1.58)	0.277	1.23 (0.95 to 1.59)	0.114
Getting on not so well					2.92 (1.89 to 4.51)	<0.001	2.89 (1.86 to 4.50)	<0.001	2.93 (2.00 to 4.28)	<0.001
**Religion (vs. Non Catholic Christianity)**										
Catholicism					1.18 (0.80 to 1.75)	0.398	1.13 (0.76 to 1.68)	0.544		
Hinduism					0.92 (0.53 to 1.57)	0.748	0.96 (0.56 to 1.66)	0.899		
Islam					0.81 (0.31 to 2.10)	0.663	0.86 (0.33 to 2.24)	0.757		
Other					1.21 (0.73 to 2.00)	0.451	1.24 (0.75 to 2.04)	0.408		
None					1.22 (0.83 to 1.82)	0.317	1.22 (0.82 to 1.83)	0.321		
**Religious attendance at 11 -13y (vs. ≥1/week)**										
Once/week					1.14 (0.78 to 1.65)	0.507	1.08 (0.74 to 1.58)	0.679		
Never					0.88 (0.56 to 1.40)	0.60	0.85 (0.54 to 1.36)	0.50		
**Religious attendance at 14 -16y (vs. ≥1/week)**										
Once/week					1.50 (1.01 to 2.23)	0.045	1.50 (1.01 to 2.25)	0.045	1.53 (1.09 to 2.14)	0.013
Never					1.90 (1.22 to 3.00)	0.005	1.88 (1.19 to 2.97)	0.007	1.87 (1.31 to 2.67)	0.001
**Parental control**^**b**^ **at 11-13y**										
Intermediate parental care tertile					0.86 (0.64 to 1.15)	0.302	0.81 (0.61 to 1.09)	0.171		
Low parental care tertile					0.78 (0.56 to 1.08)	0.135	0.79 (0.57 to 1.11)	0.175		
**Psychological distress 11-13y (TDS ≥17 vs < 17)**^**c**^					1.16 (0.82 to 1.65)	0.396	1.16 (0.82 to 1.65)	0.398		
**Psychological distress at 14-16y (TDS ≥17 vs < 17)**^**c**^					1.49 (1.05 to 2.12)	0.027	1.48 (1.04 to 2.11)	0.029	1.62 (1.17 to 2.23)	0.003
**Ethnicity (vs. White UK)**										
Black Caribbean			0.41 (0.29 to 0.57)	<0.001	0.45 (0.29 to 0.69)	<0.001	0.45 (0.29 to 0.69)	<0.001	0.47 (0.32 to 0.70)	<0.001
Black African			0.16 (0.10 to 0.25)	<0.001	0.23 (0.13 to 0.40)	<0.001	0.23 (0.12 to 0.41)	<0.001	0.25 (0.15 to 0.42)	<0.001
Indian			0.21 (0.12 to 0.36)	<0.001	0.35 (1.19 to 0.64)	0.001	0.43 (0.19 to 0.97)	0.042	0.30 (0.17 to 0.53)	<0.001
Pakistani/Bangladeshi			0.34 (0.22 to 0.52)	<0.001	0.42 (0.24 to 0.72)	0.002	0.49 (0.23 to 1.02)	0.057	0.44 (0.28 to 0.72)	0.001
Other			0.56 (0.42 to 0.73)	<0.001	0.53 (0.38 to 0.73)	<0.001	0.54 (0.38 to 0.76)	0.001	0.55 (0.40 to 0.75)	<0.001
**Born abroad (vs. born UK)**			0.57 (0.41 to 0.79)	0.001	0.65 (0.44 to 0.96)	0.047	0.67 (0.44 to 1.00)	0.049	0.56 (0.39 to 0.81)	0.002
**Family structure and parental employment at 11-13y (vs. 2 parent family, both parents employed)**										
1 parent family, 1 parent employed							1.33 (0.86 to 2.04)	0.199		
2 parent family, both parents unemployed							0.80 (0.55 to 1.45)	0.66		
1 parent family, parent unemployed							1.41 (0.88 to 2.26)	0.158		
Other family type							1.38 (0.30 to 6.30)	0.679		
**Paternal smoking 11-13y (vs. no paternal smoking)**					1.40 (1.07 to 1.82)	0.014	1.54 (1.16 to 2.05)	0.003	1.69 (1.33 to 2.15)	<0.001
**Paternal smoking at 14-16y (vs. no paternal smoking)**					1.00 (0.96 to 1.04)	0.854	0.98 (0.94 to 1.02)	0.341		
**Females (vs. males)**			1.69 (1.35 to 2.12)	<0.001	1.49 (1.05 to 2.12)	0.027	1.45 (1.11 to 1.88)	0.006	1.47 (1.14 to 1.87)	0.002

Model 0: No adjustment for covariates. Model 1: Age at both survey waves +gender+ ethnicity+ generational status. Model 2: Model 1 + religious affiliation + religious attendance at 11-13y and 14-16y + psychological distress at 11-13y and 14-16y + relationship with key parent at 11-13y and 14-16y + parental control at 11-13y + maternal and paternal smoking at 11-13y and 14-16y. Model 3 (Full Model): Model 2 + family structure and employment status at 11-13y. Model 4 (Final Model based on Wald tests of variables in model 3): Adjusted for gender, ethnicity, age, generational status, psychological distress at 14-16y + relationship with key parent at 14-16y + religious attendance at 14-16y+paternal smoking at 11-13y.

^a^ Experiences of discrimination scale which includes questions on 'unfair treatment' on the grounds of race, skin colour, place of birth and religion in various locations e.g. school, work, on the street [51].

^b^ Perceived parental care and control measured using the Parental Bonding Instrument [52]

^c^ Total Difficulties Score derived from the Strengths and Difficulties Questionnaire [53]. Score of >17 indicates psychological distress/behavioural difficulties

### Racism and ever smoking from adolescence to adulthood

S1 Table shows that at 21-23y ethnic minorities continued to be less likely to smoke, and more likely to report racism and attend a place of worship than their White British peers. As in early adolescence, Black Africans were more likely to attend a place than once per week than the other ethnic groups. The smoking patterns suggest that in adolescence females experienced a greater increase in smoking than males, but between 14-16y and 21-23y the increase was greater for males. Table 6 shows that racism remained an independent correlate of ever smoked from early adolescence to adulthood, with unadjusted effect (Model 0: odds ratio 2.93, 2.10 to 4.10) attenuating on adjustment for demographic (M1:1.98, 126 to 3.09) and psychosocial (Model 3: 1.88, 1.23 to 2.87) factors. Relationship with parent and attendance to a place of worship remained significant independent influences on smoking. The pattern of lower odd ratios for smoking among ethnic minority groups remained in early adulthood.

**Table 6:** Ever smoking at 11-23y: The influence of racism, ethnicity, parenting and religious involvement, socio-economic circumstances: Odds ratio (95% confidence interval). The Determinants of Adolescent (now adult) Social well-being and Health 10% (N = 665) pilot follow-up study.

	Model 0		Model 1		Model 2		Model 3		Model 4	
	* *	*p-value*	*demographics*	*p-value*	Model 1+ parenting, religious involvement and other psychosocial factors	*p-value*	Model 2+ Socio-economic circumstances	*p-value*	Final model	*p-value*
**Racism (vs. no racism)**^**a**^	2.93 (2.10 to 4.10)	<0.001	1.98 (1.26 to3.09)	0.003	1.88 (1.23 to2.87)	0.003	1.90 (1.24 to2.91)	0.003	1.90 (1.25 to2.90)	0.003
**Relationship with key parent (vs. getting on very well)**	** **									
Getting on quite well					1.20 (0.85 to2.00)	0.227	1.29 (0.83 to1.99)	0.252	1.29 (0.84 to1.97)	0.242
Getting on not so well					8.51 (3.02 to24.0)	0.008	8.53 (3.01 to24.2)	<0.001	8.03 (2.89 to22.3)	<0.001
**Religious attendance**	** **									
**(vs. ≥ 1/week)**	** **									
Less than once a week					1.68 (1.01 to2.80)	0.047	1.69 (1.01 to2.82)	0.047	1.61 (0.97 to2.68)	0.064
Never					2.29 (1.18 to4.47)	0.015	2.33 (1.19 to4.56)	0.015	2.41 (1.29 to4.48)	0.005
**Psychological distress (vs. no psychological distress)**^**b**^	** **				1.69 (1.02 to2.80)	0.043	1.67 (1.00 to2.78)	0.05	1.61 (0.97 to2.66)	0.064
**Ethnicity (vs. White UK)**	** **									
Black Caribbean			0.33 (0.14 to0.75)	0.008	0.52 (0.20 to1.34)	0.175	0.53 (0.20 to1.36)	0.187	0.42 (0.17 to1.05)	0.063
Black African			0.23 (0,10 to0.54)	0.001	0.44 (0.16 to1.21)	0.114	0.45 (0.16 to1.24)	0.121	0.35 (0.13 to0.91)	0.031
Indian			0.16 (0.07 to0.40)	<0.001	0.26 (0.07 to0.91)	0.036	0.26 (0.07 to0.93)	0.038	0.20 (0.07 to0.53)	0.001
Pakistani & Bangladeshi			0.23 (0.20 to0.54)	0.001	0.21 (0.06 to0.78)	0.019	0.22 (0.06 to0.82)	0.024	0.33 (0.13 to0.82)	0.017
Other			0.61 (0.27 to1.40)	0.247	0.86 (0.34 to2.20)	0.761	0.88 (0.34 to2.27)	0.795	0.76 (0.32 to1.83)	0.549
**Born abroad (vs. born UK)**	** **		0.45 (0.21 to 0.94)		0.47 (0.21 to 1.04)	0.063	0.47 (0.21 to 1.04)	0.063		
**Females (vs. males)**	** **		0.62 (0.38 to1.02)	0.063	0.56 (0.33 to0.96)	0.034	0.56 (0.33 to0.96)	0.034	0.58 (0.34 to0.98)	0.044
**Age**	** **		1.34 (1.28 to1.40)	<0.001	1.29 (1.23 to1.36)	<0.001	1.31 (1.24 to1.39)	<0.001	1.30 (1.23 to1.36)	<0.001

Model 0: No adjustment for covariates. Mode1: Adjusted for gender + age + ethnicity + generational status. Model 2: Model 1 + religious affiliation + religious attendance + psychological distress + relationship with key parent. Results for Religion; non-Catholic Christianity = reference, Catholicism 0.70 (0.32 to 1.52), Hinduism 0.68 (0.17 to 2.75), Islam 1.72 (0.63 to 4.67), Other 0.45 (0.17 to 1.20), none (0.53 to 2.50). Model 3: Model 2 + employment status. Results for Religion; non-Catholic Christianity = reference, Catholisicm 0.70 (0.32 to 1.52), Hinduism 0.67 (0.16 to 2.73), Islam 1.70 (0.62 to 4.66), Other 0.45 (0.17 to 1.19), none 1.12 (0.51 to 2.46). Results for employment status Vs. employed, unemployed 0.80 (0.49 to 1.31). Model 4 (Final model based on Wald tests for variables in model 3): Adjusted for gender, ethnicity, age, psychological distress, religious attendance and relationship with key parent

^a^ Experiences of discrimination scale [51] which includes questions on 'unfair treatment' on the grounds of race, skin colour, place of birth and religion in various locations e.g. school, work, on the street.

^b^ At age 11-16y Total Difficulties Score ≥ 17 derived from the Strengths and Difficulties Questionnaire [53]. At 21-23y derived from the 12 item General Health Questionnaire (GHQ-12) [54]. Score of ≥4 indicates psychological distress

### Qualitative findings

Table 7 shows key themes from the qualitative interviews and contextualises the statistical associations observed for parenting, religion and cultural integration.

**Table 7:** Qualitative findings, the determinants of Adult Social well-being and Health.

Quantitative findings	Qualitative findings
*Racism* Regardless of ethnicity, racism was an independent longitudinal correlate of ever smoking and smoking initiation	Covert racism: "it’s more kind of covert [..] to kind of notice why someone’s doing something you’d have to proper like really think about it and then you’re like, is it? Isn’t it? So it's like you can never be too sure if that’s the reason why someone’s done something." *Participant 32*, *female*, *Black Caribbean*, *Christian*, *A levels* Vicarious racism: "we’ll go to a shop or whatever and, like, you know, my mum, like, she’ll like ask for something, if they don’t quite understand what she says or. . . ., they’ll just look at her. And, like, sometimes the way people speak to her it really angers me." *Participant 39*, *female*, *Black African*, *Christian*, *GCSE*^*a*^ Stereotypes and low expectations: "I don't think people take Muslim women seriously, which frustrates me. They probably think we're oppressed and like we don't have a mind of our own. So I think, sometimes I think if I changed my name and applied I probably would get a different job.” *Participant 25*, *female*, *Bangladeshi*, *Muslim*, *degree*
*Positive parent-child relationships and religious involvement* Positive relationship with parents & attendance to a place of worship, regardless of religion, moderated the racism effect on ever smoking and smoking initiation across all ethnic groups	Support: "she always just, from a very young age, just, you know, told us we were all beautiful, we were all lovely, we were all very nice people [. . .], she’s said, “I don’t care what you do just so long as you’re happy [..] just do what makes you happy and I’ll support you.” Which is, you know, nice to hear, nice to feel, it kind of means you can’t fail in life." *Participant 8*, *female*, *White UK*, *no religion*, *degree* Ethnic and cultural socialisation: "I feel very Nigerian because that what I was brought up round, in a Nigerian culture, the food I eat, the mannerisms, just Nigerian influences around me. And at the same time I’ve also had British influences around me outside of my house, so yeah, best of both worlds I guess." *Participant 42*, *male*, *Black African*, *Christian*, *degree* Aspirations: "where the parents are coming from, they’re coming from backgrounds where it’s kind of like you do well and that’s always drummed into you [. . .] And so whenever you’re faced with a situation, you just have to get over it because you’ve got that inside you [. . .] I think it goes back to the racism thing, because like people have looked down on like Black people and so they feel they have to work harder to make something of themselves and therefore they have to just deal with these issues." *Participant 38*, *female*, *Black African*, *Christian*, *degree* Morals and values: “[Islam] teaches you so much about helping other people and just being there for other people and putting yourself in other people’s shoes and seeing what they go through and the difficulties in life. So it really teaches you a lot of patience and how people really struggle, and it helps you to understand." *Participant 11*, *male*, *Pakistani*, *Muslim*, *GCSE* Positive coping: "Christianity is quite a big—takes a big portion of Black people's lives so I think again it's kind of leaning against that [. . .] that kind of mentality you know, problems come, work over it, work though it and push through and just, you know, keep on holding on to your faith and what it means to you." *Participant 38*, *female*, *Black African*, *Christian*, *degree*

^a^ GCSEs (General Certificate of Secondary Education) are academic qualifications usually awarded on leaving secondary school at age 15-16y, dependent on successful completion of examinations

### Perceptions of racism

Qualitative accounts highlighted pervasive exposure to racism from childhood including bullying, insults, negative media stereotyping and, rarely, physical assault. Narrative accounts included experiences not captured in the self-report questionnaire such as vicarious and anticipatory racism. Racism was most commonly perceived as hidden or 'covert' than 'full-on' or 'direct', attributed to increasing diversity and social unacceptability. Commonly reported responses involved minimising the impact and avoiding confrontation through ‘ignoring’, 'brushing it off', or humour. Some Muslim participants felt that explicit racism had increased in recent years due to changes in public attitudes associated with political events and terrorist attacks. Some participants described discrimination by teachers/lecturers who they felt had lower expectations of them in terms of educational achievement and career prospects. Black Caribbean and African participants and Muslim women in particular expressed anxiety about the impact of stereotypes which they feared might lead to discrimination when seeking employment.

### Parental support, socialisation and aspirations

Tactics for dealing with racism were generally not explicitly discussed with parents and tended to be more defensive than confrontational. Indirect parental influences on coping were attributed to, for example, observing parents' forbearance in the face of hardship, including racism, to achieve future life goals. Securing high status careers such as doctors or lawyers was seen as potentially buffering the impact of racism, though some expressed expectations that ethnic minorities needed to 'work harder' than the majority population to overcome structural disadvantages and low expectations. Parents were reported to foster ethnic and cultural socialisation, imparting a shared sense of 'our culture' and associated values and practices, such as family loyalty, high aspirations and a work ethic. At the same time many parents were also reported to instil a 'British' identity and an ability to 'mix into any community'.

### Religious faith and values

Personal faith and religious values continued to influence lifestyles and coping in young adulthood, regardless of the extent of formal religious practice. This varied from a belief in God as a source of comfort and self-affirmation, to a more active coping style based on prayer. Attendance to places of worship and participation in religious practices were commonly discussed as consolidating parental roles in identity formation and instilling morals and values. These included an ethic of tolerance and endurance, a sense of meaning, purpose and self-worth, and positive coping strategies in the face of adversity, including racism. Several participants, particularly Africans and Muslims, continued regular involvement with a place of worship in adulthood. A few described 'turning points' where they had developed their own religious practice as adults which led to lifestyle changes and a sense of purpose.

### Cultural integration

The quantitative results did not suggest a consistent relationship between cultural integration and smoking. However, responses from qualitative interview participants suggested that the diversity of London neighbourhoods, schools and workplaces may have discouraged overt racism while providing opportunities for developing ethnic, religious or cultural solidarities. Such diversity could foster a sense of belonging, regardless of ethnicity. Participants commonly described plural or transecting identities which combined ethnic, cultural and religious identities with the rights and entitlements of British citizenship. However for some Pakistani and Bangladeshi participants high density 'Asian' or 'Muslim' neighbourhoods were seen to protect against racist encounters.

Though inter-ethnic friendships were described, cultural differences in values and lifestyles could result in similar ethnic or religious groups 'sticking together':

"mostly people kept within their kind of groups. I don’t think … it wasn’t intentional, there was very little, I wouldn’t think there was any racism in our school pretty much, nothing I would have noticed really. But it just, kind of, I think it’s more what your family does, what you do, where you hang out, what you do after school, kind of thing. And you tend to follow with your kind of ethnicity, as it were" *Participant 8*, *female*, *White British*, *no religion*, *degree*

Others spoke about the value of 'branching out' from one's 'comfort zones' and 'mixing' with people of other ethnic or religious groups to develop adaptable social skills and counter stereotypes:

"for her [White British friend] it wasn’t any form of racism, it was just like she hasn’t grown up with anything apart from what she knows and so for her it would be just “weird”, I was like: “It’s not weird, it’s just different.” So I think the more people mix the more you get to understand her culture and she gets to understand my culture" *Participant 30*, *female*, *Black African*, *Christian*, *degree*

Whilst ‘sticking together’ could reinforce culturally proscribed behaviours, such as not smoking, ‘mixing’ could lead to experimentation with behaviours which were contrary to cultural/religious norms, such as drinking alcohol and smoking.

## Discussion

This is the first known longitudinal study of racism and smoking in an ethnically diverse UK cohort. Racism had a powerful independent impact on smoking regardless of ethnicity, socio-economic disadvantage and parental smoking. Ethnic or gender specific effects of racism on smoking were not evident in these analyses. Parenting and religious involvement buffered the impact of racism on smoking behaviour. Qualitative findings suggested that these factors may have operated through aspects of social support, ethnic/cultural socialisation, the instillation of morals and values, educational and career aspirations, and positive coping styles. The findings signal an emerging public health concern particularly for some ethnic minorities in the UK, among whom smoking levels have been historically low. While other studies have shown that White British and Black Caribbean young people are more likely to smoke than Asian or Black African groups [58–60], in our study, smoking increased across all ethnic groups and genders from early adolescence to adulthood.

### Racism, smoking and sociocultural buffers

The results, shown for the first time for the UK, strengthen the predominantly US evidence base on the impact of racism on adolescent smoking behaviour [16]. They lend support to biopsychosocial theories of racism triggering a stress response which is expressed in risk behaviours. Within this model coping resources and appraisal of the perceived threat, may moderate the stress response [61]. Multiple forms of discrimination over the life course may lead to accumulated disadvantage and exacerbate adverse health effects [25]. Vigilance against possible racism may also induce chronic stress [27]. The increase in reported racism with age also parallels longitudinal findings from the US [62]. Reported racism in DASH is relatively high (~50% reporting racism at 21-23y) compared to the 20–25% of UK ethnic minority adults reporting racism [63]. This may reflect actual rises in racist attitudes and behaviour and/or the impact of historical events such as the London bombings of 2007 which occurred during follow-up at 14-16y and led to a rise in anti-Islam sentiment.

The influence of parenting on smoking is consistent with evidence that quality of parent-child relationships influences engagement with risk behaviour, including smoking [37]. Supportive parenting may also buffer racism through nurturing self-esteem and the acquisition of positive coping strategies [11]. As shown in the qualitative interviews, parental relationships can play an important role in 'ethnic socialisation' and development of a positive cultural identity [41]. Religious attendance appeared to be an independent deterrent to smoking initiation in late adolescence and to continuation of smoking from childhood to adulthood. Affiliation to a particular religion was less important than religious attendance. Other studies have shown some evidence for the protective effect of religious involvement on health behaviours in adolescence [64], however only a minority use longitudinal data making it difficult to examine the impact of religion over the life course or disentangle the effects of attendance from religious affiliation. Religion embraces social, cultural and psychological dimensions which can be difficult to capture using standardised measures. Qualitative methods, as shown in this study, can shed light on the ways through which aspects of religion may intersect with other aspects of culture, identity and family life to buffer adversity and promote healthy behaviours across different religions, genders and ethnicities, beyond social support and religious norms.

The ethnic patterning of tobacco use and the significance of family life and religious involvement suggest that sociocultural factors retain an important influence on smoking behaviour. These may play a stronger role in relation to smoking compared to other health behaviours due to moral sanctions which may not operate in relation to diet for example. The lower rates of smoking in South Asian girls has been replicated in other studies [59] consistent with a strong gender effect in this group. Cultural and religious disapproval of smoking may be particularly significant for South Asian girls who may be more subject to community sanctions on behaviour compared to boys [65]. The lower rates of smoking among those born abroad compared to those born in the UK shows some convergence to the smoking habits of the majority population, as shown in other studies [58]. Friendships with those of similar ethnicity or religion are likely to enhance 'bonding' social capital and reinforce adherence to cultural norms, whereas friendships across cultures may challenge such norms through greater exposure to alternative lifestyles, as in the 'acculturation' hypothesis [12]. However a combination of 'cultural maintenance' alongside engagement with wider society may foster resilience to factors which can increase the likelihood of smoking, such as racism and peer pressure, through enhancing both ‘bonding’ and 'bridging' social capital and providing a broader repertoire of psychosocial skills and coping strategies [12, 38].

### Strengths and limitations

Measures of racism and discrimination vary which limits comparison with other studies. As with other multi-dimensional concepts, although we used a measure which has been widely used in the US and elsewhere, standardised measures of racism and discrimination are subject to challenges in validity and interpretation [48]. We aimed to mitigate this at age 21-23y through the addition of qualitative interviews with a sub-sample. They provided some insight into the specific contextual and conceptual interpretations of racism, and the intersections of family life, religion and ethnic identity which might mitigate the impact. DASH is a London-based study and the experiences of ethnic minorities in a large diverse methropolitan city are probably not generaliseable to all parts of the UK, particularly rural areas or those with little ethnic diversity. That said, a key strength of DASH is its diverse sample, high retention rates and low item-non-response, mainly due to strong support from local communtities. Smoking, often underestimated in young people's self-reports, was validated by salivary cotinine in a sub-sample at ages 11–13 years [66]. Ethnic specific effects were not evident from interaction terms (ethnicity x racism) but this may have been due to the lack of statistical power from small sample sizes. Analyses stratified by ethnicity, though limited by small sample sizes, suggested similar effects of racism within the ethnic groups and supported the interpretation of the analyses using interaction terms in that the effect sizes were similar. We did not ask about the use of water pipes which are reportedly used more than cigarettes by South Asians in London [67], and thus use of tobacco may have been under-reported in this group. The different age trends by gender for smoking, however, are consistent with statistics for England over the same time period (2002–14), with more males than females over age 16y smoking [1]. The influence of culturally patterned psychosocial influences, independent of SEC, might have been amplified without adjustment for early childhood and/intergenerational measures of SEC. In early adolescence most participants were unaware of their parents’ education level or occupation.

## Conclusion

These findings highlight the role of racism in sustaining health inequalities and the need to address racism as an important social determinant of health within 'whole system' approaches which include families, communities, health and social services, and the wider political and economic context. Failing to consider the impact of discrimination and disadvantage on health behaviours such as smoking, where the social gradient remains significant, means that preventive strategies are likely to widen inequalities in health [68] as shown for indigenous/first nation groups in Australia, New Zealand, Canada, and the US [69], as well as young people from economically disadvantaged backgrounds in the UK [1]. Parental care and religious involvement may act as forms of social capital to moderate the impact of economic deprivation and racism on health and well-being, including health risk behaviours, through facilitating access to social support and resources and nurturing a sense of identity and belonging [37]. However addressing structural discrimination, as highlighted in the recent UK government Race Disparity Audit, and ensuring equity of opportunity in education and employment, for example, is vital to enable such assets to be potentiated. Ethnic and religious penalties in employment outcomes for young adults [70] and the consequences for health across the life course reinforce the need for continued efforts to combat racism and discrimination at all levels in the interests of equity and health.

## Supporting information

S1 Table Characteristics of the DASH sample at 21-23y (N = 665) by ethnicity (%). The Determinants of Adolescent (now Adult) Social well-being and Health.
(PDF)

S1 Appendix Qualitative interviews topic guide. (PDF)
