# Supplementary material for: The influence of racism on cigarette smoking: Longitudinal study of young people in a British multiethnic cohort
Source: PLoS One. 2018 Jan 24;13(1):e0190496. doi: 10.1371/journal.pone.0190496 (PMC5783341; doi:10.1371/journal.pone.0190496)
Supplement: S1 Table — The Determinants of Adolescent (now Adult) Social well-being and Health. (PDF) [file pone.0190496.s001.pdf]

S1 Table: Characteristics of the DASH sample at 21-23y (N=665) by ethnicity (%).The Determinants of Adolescent (now Adult) Social well-being and Health

	**White UK (N=107)**	**Black Caribbean (N=102)**	**Black African (N=132)**	**Indian (N=99)**	**Pakistani/Bangladeshi (N=111)**	**All (N=665)**
**Own smoking**						
Ever smoked	75.7 (66.6 to 82.9)	51.0 (41.3 to 60.6)*	41.7 (33.5 to 50.3)*	44.4 (34.9 to 54.4)*	42.3 (33.4 to 51.7)*	55.3 (46.0 to 64.2)*
Ever smoked (Males)	77.0 (63.4 to 86.5)	48.9 (34.9 to 63.1)*	45.6 (33.1 to 58.7)*	53.8 (40.2 to 67.0)*	53.8 (40.2 to 67.0)*	57.9 (44.7 to 70.1)*
Ever smoked (Females)	74.5 (61.3 to 84.4)	52.7 (39.5 to 65.6)*	38.7 (28.3 to 50.2)*	34.0 (21.9 to 48.8)*	38.8 (26.1 to 53.1)*	52.6 (39.6 to 62.3)*
**Racism**[a]						
Reported racism[a]	19.6 (13.1 to 28.3)	48.0 (38.5 to 57.7)*	51.5 (42.0 to 60.0)*	38.4 (29.3 to 48.4)*	46.8 (37.7 to 56.2)*	50.9 (41.7 to 60.0)*
**Psychosocial factors**						
Parenting						
Getting on very well with key parent	66.3 (56.8 to 74.7)	61.8 (51.9 to 70.7)	56.1 (47.4 to 64.3)	66.6 (54.7 to 73.5)	62.2 (52.7 to 70.7)	65.8 (56.6 to 73.9)
Getting on not so well with key parent	0.9 (0.13 to 6.40)	2.9 (0.94 to 8.80)	5.3 (2.54 to 10.75)	3.0 (0.97 to 9.0)	3.6 (1.35 to 9.3)	3.5 (1.30 to 9.0)
Religion						
Non Catholic Christianity	24.3 (17.1 to 33.4)	58.8 (49.0 to 68.0)*	59.1 (50.5 to 67.2)*	7.1 (3.4 to 14.1)*	-	15.8 (10.1 to 23.7)
Catholicism	9.3 (5.1 to 16.6)	25.5 (17.9 to 34.9)*	20.4 (14.4 to 28.2)*	4.0 (1.5 to 10.3)*	-	21.0 (14.5 to 29.5)
Hinduism	-	-	-	52.0 (42.7 to 62.2)	-	1.7 (0.40 to 6.8)
Islam	-	-	9.8 (5.8 to 16.3)	18.2 (11.7 to 27.1)	91.9 (85.1 to 95.7)	23.7 (16.7 to 32.4)
Other	5.6 (2.5 to 12.0)	2.9 (0.9 to 8.8)	3.0 (1.1 to 7.8)	9.1 (4.8 to 16.6)	-	8.8 (4.8 to 15.6)
None	52.3 (42.8 to 61.7)	6.9 (3.3 to 13.8)*	-	1.0 (0.14 to 6.9)*	-	19.3 (13.0 to 27.6)**
Religious attendance						
Attendance at a place of worship ≥1x per week	2.0 (0.5 to 7.8)	24.0 (16.4 to 33.6)*	52.1 (43.1 to 61.0)*	25.8 (17.8 to 36.0)*	44.3 (34.7 to 54.4)*	18.9 (12.5 to 27.5)*
No attendance at a place of worship	72.7 (63.1 to 80.6)	33.3 (24.6 to 43.4)*	10.1 (5.8 to 17.0)*	18.0 (11.3 to 27.4)*	21.6 (14.5 to 30.0)*	50.9 (41.4 to 60.4)*
Generational status						
Born in the UK	97.2 (91.7 to 99.1)	86.3 (78.1 to 91.7)*	71.2 (62.9 to 78.3)*	81.8 (72.9 to 88.3)*	82.0 (73.6 to 88.1)*	75.4 (66.7 to 82.5)*
Born abroad	0.90 (0.12 to 6.4)	9.8 (5.3 to 17.3)	28.0 (21.0 to 36.3)*	18.2 (11.7 to 27.1)*	16.2 (10.4 to 24.3)*	21.9 (15.2 to 30.5)*

Percentages do not add up to 100% due to missing values.

[a] Experiences of discrimination scale which includes questions on 'unfair treatment' on the grounds of race, skin colour, place of birth and religion in various locations e.g. school, work, on the street [51]

[b] Derived from the 12 item General Health Questionnaire (GHQ-12). Score of ≥4 indicates psychological distress [54]
