# Supplementary material for: The influence of racism on cigarette smoking: Longitudinal study of young people in a British multiethnic cohort
Source: PLoS One. 2018 Jan 24;13(1):e0190496. doi: 10.1371/journal.pone.0190496 (PMC5783341; doi:10.1371/journal.pone.0190496)
Supplement: S1 Appendix — (PDF) [file pone.0190496.s002.pdf]

Appendix 1: Qualitative interviews topic guide

1. Socio-economic circumstances:

1.1 Educational history and qualifications

1.2 Employment/study

- Nature of employment/study
- Working hours and conditions - permanent/temporary; full-time/part-time; salaried or hourly rate
- Satisfaction with work/study
- Experience of finding work, prior unemployment
- Areas of difficulty/success
- Relationships with students/workmates
- Experiences of discrimination
- How far work/study matches with childhood aspirations
- Influence on occupational choices – parents, family, friends, school, culture
- Future plans

1.3 Other regular activity – hobbies, voluntary work

1.4 Housing

- Tenure, cost, contribution to rent
- How long he/she has lived in property
- Number of people living in the same accommodation and relationship (family, friends, co-tenants)
- Condition of the property (participant report/researcher's observation)
- How many rooms, own room
- Likes and dislikes about accommodation – cost, overcrowding

1. 5 Finances

- Sources of income – salary, student loan, benefits, support from family
- Financial difficulties, e.g. debt
- Contribution to household budget

2. Health

2.1 General health

- How the participant feels health is generally
- Check for previous or current conditions
- In receipt of treatment – NHS and/or other

2.2 Mental health

- How the participant feels mental health is generally

- Check for previous or current conditions
- In receipt of treatment – NHS and/or other

2.3 Explore any particular stresses the participant is facing in life
e.g. work, relationships, money, discrimination
Elicit an example if possible.

2.4 Explore opportunities/challenges in transition from teens to adulthood and feelings about these.

- Responsibilities, decision-making
- Finances
- Changes in relationships – family/friends/colleagues/romantic
- Areas of stress or conflict

2.5 Explore what helps the participant to cope with difficulties/changes in life.
e.g. friends, family, religion
If possible, elicit an example of when the person faced a problem and how they responded.

3. Identity and discrimination

3.1 Ethnic identity

- How the participant defines ethnicity – to self/others
- How strongly he/she identifies with ethnicity - visits to 'home' country, use of another language, food, music, clothing, membership of any ethnically affiliated organisations
- Experiences of racism/discrimination – media, society, community, school, workplace

3.2 Religion

- How participant defines his/her religious affiliation
- Nature and frequency of attendance at place of worship and religious practice
- Importance of belief/religious identity
- Experiences of discrimination - media, society, community, school, workplace

3.3 Gender

- Gender identity
- Social/cultural expectations of role
- Experiences of discrimination - media, society, community, school, workplace

4. Relationships

4.1 Family relationships

- amount of contact
- level of support
- helpful/unhelpful aspects of family relationships
- areas of agreement and conflict

4.2 Friendships

- amount of contact
- level of support
- mainly from own or other ethnic groups
- areas of agreement and conflict

4.3 Romantic relationships

- how long together
- amount of contact
- level of support
- from own or other ethnic group
- areas of agreement and conflict

5. Neighbourhood environment

- feelings about neighbourhood e.g. sense of community/belonging, crime/safety, expense
- relationships with neighbours
- use of community resources

6. Political and economic situation

- impact of government policy on participant, changes to student finances/benefits
- impact of economic downturn
- government policy/societal attitudes towards ethnic minorities/immigration
